# Emergency Pancreaticoduodenectomy in Duodenal Paraganglioma: Case Report

**DOI:** 10.5402/2011/268674

**Published:** 2011-04-12

**Authors:** Fazl Q. Parray, Iqbal M. Lone, Nisar A. Chowdri, Imtiaz Wani, Mehmood A. Wani, G. M. Gulzar, Natasha Thakur

**Affiliations:** ^1^Department of General Surgery, SKIMS, Srinagar, Kashmir 190011, India; ^2^Department of Pathology, SKIMS, Srinagar, Kashmir 190011, India; ^3^Department of Gastroenterology, SKIMS, Srinagar, Kashmir 190011, India

## Abstract

Duodenal gangliocytic paraganglioma (DGP) is a rare tumor that characteristically occurs in the second part of duodenum. These appear as submucosal masses that protrude into the lumen of a duodenum. Gastrointestinal bleeding is the commonest manifestation of DGP. Metastatic spread to regional lymph nodes occurs rarely. Surgical resection is the treatment of choice for DGP. A case of a DGP is reported in young female who presented with a recurrent upper gastrointestinal bleeding. Upper gastrointestinal endoscopy (UGIE) documented a mass in the ampullary region with ulceration in its middle which was bleeding. Recurrent gastrointestinal bleeding necessitated an emergency pancreaticoduodenectomy. Histopathology of specimen documented gangliocytic paraganglioma.

## 1. Introduction

Duodenal gangliocytic paraganglioma (DGP) was first described by Dahl et al. [1] in 1957 and subsequently reported by Taylor and Helwig [2] in 1962. In 1971, the Kepes and Zacharias [3] named the tumor and described its characteristics. DGP is a rare gastrointestinal tumor that is found primarily in the second part of duodenum. These have a variable presentation, sometimes detected incidentally. Approximately 5% of cases of DGP demonstrate malignant features [4]. Gangliocytic paragangliomas exhibit characteristic triphasic microscopic appearance with epithelioid cells, spindle cells, and ganglion cells resulting in a complex histology with features of paraganglioma, carcinoid, and ganglioneuroma. Resection of tumour is modality of treatment.

## 2. Case Report

A 20-year-old female presented with passage of black tarry stools. On general physical examination, she had an anxious look, pallor, tachycardia of 98/min, and B.P of 100/70 mm Hg. per abdominal examination was normal. Digital per rectal examination confirmed malena. She had resuscitation and the transfusion of 3 units of packed red cells. Emergency upper gastrointestinal endoscopy (UGIE) revealed fleshy exophytic sub mucosal tumor with ulceration in its middle arising in the second part of a duodenum. A biopsy was taken which was reported as an adenocarcinoma. Hemoglobin of 8.3 g/dL and hematocrit of 40% was present. A mass in the ampullary region was documented on abdominal sonography. CECT scan (contrast-enhanced computed tomography) abdomen revealed a mass in periampullary region with no evidence of lymphadenopathy or any metastasis. On the fourth day of admission, patient had another episode of a massive UGI bleed (Malena) and had the transfusion of three more units of packed red cells. 14 hours after the last episode of UGI bleeding patient had another episode of a malena with hematemesis. Four more units of packed red cells were transfused. After resuscitation, patient had emergency surgical exploration. Pylorus preserving pancreaticoduodenectomy in view of an ampullary mass (adenocarcinoma) was done. No regional lympahadenopthy was present. A polypoid mass measuring 5.0 × 3.2 × 1.5 cms. was seen arising from the second part of duodenum with ulceration in its centre.

**Figure 1:**
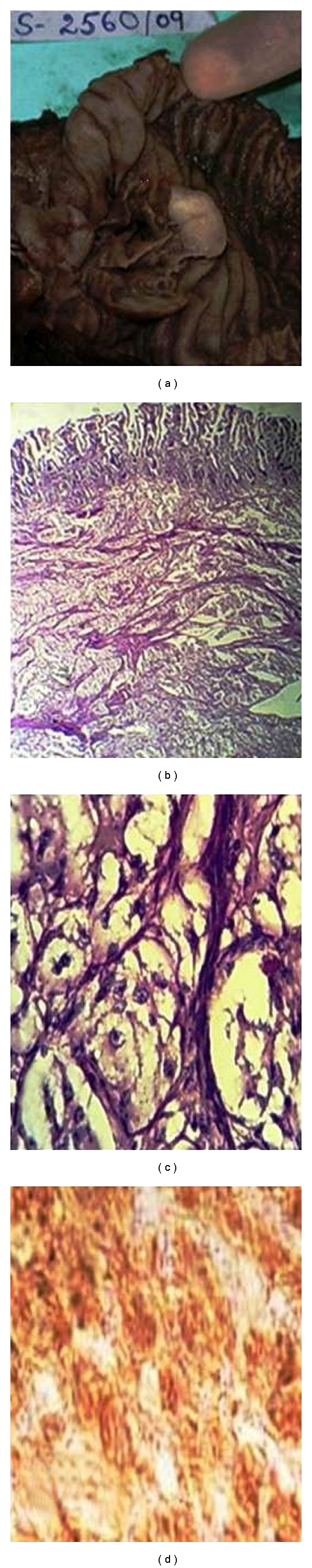
Gangliocytic paraganglioma, (a) grey-white, homogenous cut surface of intraluminal polypoid growth. (b) Low-power view with distortion of duodenal glands by sub mucosal tumor in organoid pattern. (c) High-power view showing spindle cells and ganglion cells with pale nuclei and prominent nucleoli. (d) Tumor having immunoreactivity for synaptophysin.

On cut section, the tumor was tan, white, and yellow and was well demarcated from the normal surrounding tissue (Figure 1(a)). There was no gross evidence of capsule formation. Postoperative period was uneventful. Microscopically, the tumor was well circumscribed without capsule formation. The tumor was present in the submucosa and invading the lamina propria and muscularis propria. There were 3 cell types: epithelioid cells, surrounding spindle-shaped sustentacular cells, and scattered ganglion cells. The epithelioid cells were arranged in nests displaying round nuclei with stippled chromatin, small yet conspicuous nucleoli, and delicate granular cytoplasm. These spindle cells were elongated and occasionally plump, with granular chromatin and moderate amounts of elongated, eosinophilic cytoplasm. Ganglion cells were large with prominent nuclei and nucleoli and had abundant eosinophilic cytoplasm.

No cytomorphologic atypia was present. Necrosis and mitotic figures were not present (Figures 1(b) and 1(c)). Immunohistochemical staining showed that the ganglionic cells were positive for synaptophysin (Figure 1(d)). Diagnosis of duodenal paraganglioma was confirmed on histopathology. Patient had followup for more than 11 months with no evidence of recurrence.

## 3. Discussion

DGP is a rare neuroendocrine tumour. The commonest site of origin is the second part of a duodenum, follwing duodenum in gut this can arise from jejunum and pylorus. Lung, bronchus, appendix, mesentery, and nasopharynx are other rare sites in body where gangliocytic paraganglioma can arise [5, 6]. Age of presentation is 15 to 80 years and is usually seen in a sixth decade of life. DGP usually measures between 1 and 3 cms. in maximum diameter, although in rare instances lesions measuring 10 cms. in maximum diameter have been also reported.

Cell of origin of gangliocytic paraganglioma is an embryonic celiac ganglion or from pluripotent stem cells located at the base of the intestinal glands [2]. Hamartomatous proliferation of endodermally derived epithelial cells originating from the ventral primordium of the ventral pancreas and neuroectodermally derived ganglion and schwanian cells has also been suggested as an origin of this tumor [7]. Common presenting symptoms of DGP are gastrointestinal bleeding, abdominal pain, and occasionally biliary obstruction. Gastrointestinal bleeding is due to mucosal ulceration. Biliary obstruction manifests as an obstructive jaundice, this manifestation is rare to see [8]. In some unusual circumstances, duodenal gangliocytic paraganglioma has been reported to present as duodenal obstruction [9]. These duodenal tumours may remain asymptomatic and sometimes detected incidentally. There had been, in general, no known association between duodenal paraganglioma and other diseases; although association of paraganglioma and neurofibromatosis, and the paraganglioma and duodenal adenocarcinoma has been reported in the literature [10, 11]. UGIE shows DGP as a sessile or polypoid submucosal lesion with normal overlying mucosa with or without ulceration. DGP are centered on the sub mucosa which invariably is responsible for a negative endoscopic biopsy as was seen in our case. On computed tomography, it appears as an intensely enhancing mass in the region of the pancreatic head, and this intense enhancement rules out adenocarcinoma and focal pancreatitis [12]. 

This tumor consists of a complex neoplastic infiltration with a component resembling carcinoid or islet cell tumor, admixed with proliferation of spindled neurofibrillary cells and larger polygonal cells demonstrating gangliocytic differentiation. There may be stromal hyalinization in some cases. Cellular pleomorphism may be prominent, but mitoses and necroses are rare. Previously, it was reported that documented lymph node metastasis has epithelioid cells, but there are reports that metastatic deposits of tumor in regional lymph nodes have the DGP components (spindle cells, ganglion-like cells, and epithelioid cells) [13].

Gangliocytic paraganglioma are considered benign, and treatment is complete excision of tumor. Surgical or an endoscopic resection can be used for excision. Careful assessment prior to local excision is necessary. In case of diagnostic dilemma, lesion is large, or there is a suspicion of lymph node involvement, pancreaticoduodenectomy with lymph node dissection is recommended. For those who had local resection only, meticulous followup is mandatory. Transduodenal laparoscopic resection with intraoperative duodenoscopy is a valuable treatment for benign gangliocytic paraganglioma of the duodenum which is unresectable by upper gastrointestinal endoscopy [14]. Recently intensity-modulated radiotherapy adjuvant treatment strategy has been determined in cases that demonstrate regional or distant metastasis [4].
